# Development of Echinocandin Resistance in *Candida haemulonii*: An Emergent, Widespread, and Opportunistic Fungal Pathogen

**DOI:** 10.3390/jof9080859

**Published:** 2023-08-18

**Authors:** Laura N. Silva, Lívia S. Ramos, Simone S. C. Oliveira, Lucas B. Magalhães, Jefferson Cypriano, Fernanda Abreu, Alexandre J. Macedo, Marta H. Branquinha, André L. S. Santos

**Affiliations:** 1 Laboratório de Estudos Avançados de Microrganismos Emergentes e Resistentes (LEAMER), Instituto de Microbiologia Paulo de Góes (IMPG), Universidade Federal do Rio de Janeiro (UFRJ), Rio de Janeiro 21941-902, Brazil; lauransilva@gmail.com (L.N.S.); liviaramos2@yahoo.com.br (L.S.R.); simonesantiagorj@yahoo.com.br (S.S.C.O.); lbarrosmagalhaes@gmail.com (L.B.M.); mbranquinha@micro.ufrj.br (M.H.B.); 2 Laboratório de Biologia Celular e Magnetotaxia & Unidade de Microscopia Multiusuário, Instituto de Microbiologia Paulo de Góes (IMPG), Universidade Federal do Rio de Janeiro (UFRJ), Rio de Janeiro 21941-902, Brazil; jeffcy@micro.ufrj.br (J.C.); fernandaaabreu@micro.ufrj.br (F.A.); 3 Laboratório de Biofilmes e Diversidade Microbiana, Centro de Biotecnologia e Faculdade de Farmácia, Universidade Federal do Rio Grande do Sul (UFRGS), Porto Alegre 90010-150, Brazil; alexandre.macedo@ufrgs.br; 4 Programa de Pós-Graduação em Bioquímica (PPGBq), Instituto de Química (IQ), Universidade Federal do Rio de Janeiro (UFRJ), Rio de Janeiro 21941-853, Brazil; 5 Rede Micologia RJ—Fundação de Amparo à Pesquisa do Estado do Rio de Janeiro (FAPERJ), Rio de Janeiro 21941-902, Brazil

**Keywords:** non-*albicans Candida* species, virulence, *Galleria mellonella*, caspofungin, biofilm, fitness

## Abstract

Echinocandins, used for the prevention and treatment of invasive fungal infections, have led to a rise in breakthrough infections caused by resistant *Candida* species. Among these species, those belonging to the *Candida haemulonii* complex are rare multidrug-resistant (MDR) yeasts that are frequently misidentified but have emerged as significant healthcare-associated pathogens causing invasive infections. The objectives of this study were to investigate the evolutionary pathways of echinocandin resistance in *C. haemulonii* by identifying mutations in the *FKS1* gene and evaluating the impact of resistance on fitness. After subjecting a MDR clinical isolate of *C. haemulonii* (named Ch4) to direct selection using increasing caspofungin concentrations, we successfully obtained an isolate (designated *Ch4′r*) that exhibited a high level of resistance, with MIC values exceeding 16 mg/L for all tested echinocandin drugs (caspofungin, micafungin, and anidulafungin). Sequence analysis revealed a specific mutation in the resistant *Ch4′r* strain, leading to an arginine-histidine amino acid substitution (R1354H), occurring at the G4061A position of the HS2 region of the *FKS1* gene. Compared to the wild-type strain, *Ch4′r* exhibited significantly reduced growth proliferation, biofilm formation capability, and phagocytosis ratio, indicating a decrease in fitness. Transmission electron microscopy analysis revealed alterations in cell wall components, with a notable increase in cell wall thickness. The resistant strain also exhibited higher amounts (2.5-fold) of chitin, a cell wall-located molecule, compared to the wild-type strain. Furthermore, the resistant strain demonstrated attenuated virulence in the *Galleria mellonella* larval model. The evolved strain *Ch4′r* maintained its resistance profile in vivo since the treatment with either caspofungin or micafungin did not improve larval survival or reduce the fungal load. Taken together, our findings suggest that the acquisition of pan-echinocandin resistance occurred rapidly after drug exposure and was associated with a significant fitness cost in *C. haemulonii*. This is particularly concerning as echinocandins are often the first-line treatment option for MDR *Candida* species.

## 1. Introduction

Echinocandins are the newest class of antifungal agents widely used in medical clinics. Echinocandins are effective against various yeasts (e.g., *Candida* spp.) and filamentous fungi (e.g., *Aspergillus* spp.), making them suitable for the treatment of invasive infections [1,2]. Unlike amphotericin B and azoles, echinocandins—caspofungin, micafungin, anidulafungin and rezafungin—target the cell wall by inhibiting the enzyme responsible for the synthesis of β-(1,3)-d-glucan, a crucial polysaccharide in the fungal cell wall. The disturbance in glucan synthesis leads to osmotic imbalance, impairing fungal viability. β-(1,3)-d-glucan synthases are encoded by the *FKS1* gene in *Candida albicans*, *Cryptococcus neoformans*, and *Aspergillus fumigatus* and by both the *FKS1* and *FKS2* genes in *Candida glabrata* and *Saccharomyces cerevisiae* [3,4]. In *C. albicans* and certain non-*albicans Candida* species, resistance mutations occur in two highly conserved regions of the *FKS1* gene or their equivalent regions in the *FKS2* gene in *C. glabrata*. These amino acid substitutions reduce the enzyme’s sensitivity to the echinocandins, resulting in 5- to 100-fold increased MIC values [3,4]. For instance, *Candida parapsilosis* species complex and *Candida guilliermondii* exhibit higher MIC values compared to other susceptible *Candida* species due to the polymorphisms in the hot spot regions of the *FKS* gene [4]. Notably, the epidemiological shifts in infections caused by non-*albicans Candida* species, which are inherently resistant or rapidly develop resistance to azoles, have resulted in the widespread utilization of echinocandins and subsequently led to the emergence of resistant strains to this new antifungal class [3]. While some studies report relatively low frequencies of echinocandin-resistant fungal isolates, particularly for *Candida* spp. (<3%) [5,6], this percentage varies across different geographical regions and can reach nearly 12% of *Candida* isolates [7,8].

Species belonging to the *Candida haemulonii* complex (*C. haemulonii sensu stricto*, *C. duobushaemulonii*, and *C. haemulonii* var. *vulnera*) have emerged as opportunistic fungal pathogens, causing invasive infections with high rates of treatment failure [9]. Although distantly related to *C. albicans*, the *C. haemulonii* complex is closely related to other often multidrug-resistant (MDR) species such as *C. auris*, *C. pseudohaemulonii*, and *C. lusitaniae* [10]. Outbreaks in hospital settings have been reported, leading to high mortality associated with these fungal infections [11,12]. Clinical isolates of the *C. haemulonii* complex typically demonstrate resistance to first-line antifungal agents (e.g., fluconazole and amphotericin B), and they may exhibit variable susceptibility to novel azoles (e.g., voriconazole) and echinocandins [9,13]. Data suggest that drug efflux pumps play a role in mediating azole resistance, and mutations in the *ERG11* gene, which encodes the target enzyme lanosterol 14α-demethylase involved in ergosterol biosynthesis, may also contribute to resistance [14,15]. Regarding amphotericin B, low ergosterol content, dysfunctional mitochondrial properties, and robust antioxidant mechanisms controlling redox homeostasis have been implicated in its fungicidal effects and may explain the resistance observed in MDR species of the *C. haemulonii* complex [16]. However, our understanding of acquired resistance mechanisms in this emerging fungal complex remains incomplete.

Since infections caused by species belonging to the *C. haemulonii* complex have shown a limited response to azoles and amphotericin B, as described previously, echinocandins are often recommended as a first-line treatment option. However, resistance to echinocandins can develop during therapy and is associated with prolonged or repeated drug exposure, although it can also occur after brief drug exposure [1]. Therefore, it is worth investigating whether continuous exposure to echinocandin agents can induce resistance in *C. haemulonii*. In the present study, we aimed to characterize the acquired resistance in *C. haemulonii* by generating an in vitro echinocandin-evolved strain. The resistant strain of *C. haemulonii* was obtained by exposing it to increasing concentrations of caspofungin. Following the directed evolution experiment, we conducted virulence assays using in vitro interaction with macrophage cells and *Galleria mellonella* larvae as an in vivo model. Sequence analysis of the target *FKS* gene was performed to better understand the contribution of possible mutations to the acquisition of resistance. Additionally, phenotypic assays, including growth proliferation, biofilm formation capability, resistance to stress-inducing agents, and transmission electron microscopy, were employed to assess the fitness cost associated with echinocandin resistance acquisition.

## 2. Materials and Methods

### 2.1. Fungal Strain and Culture Conditions

The *C. haemulonii* strain LIP*Ch*4 (Ch4), which was isolated from a patient’s fingernail at a Brazilian hospital, was used in this study [17]. This specific fungal strain was chosen due to its proven resistance to amphotericin B (MIC = 4 mg/L), fluconazole (MIC > 64 mg/L), voriconazole (MIC > 16 mg/L), itraconazole (MIC > 16 mg/L), and posaconazole (MIC = 8 mg/L), while exhibiting susceptibility to caspofungin and micafungin with MIC values equal to 0.5 mg/L for both echinocandin drugs [15,16,17]. Fungal cells were cultivated in Sabouraud-dextrose medium (SAB) at 37 °C for 48 h with constant agitation at 120 rpm. Then, the yeasts were collected by centrifugation (4500 rpm for 5 min), washed three times in phosphate-buffered saline (PBS; pH 7.2), and suspended in the same buffer. The yeast cells were quantified using a Neubauer chamber.

### 2.2. Generation of the Echinocandin-Evolved C. haemulonii Strain

A fungal suspension containing 1 × 10^8^ yeasts in 1 mL of PBS was streaked onto SAB plates containing progressively increasing concentrations of caspofungin as proposed by the protocol published by Bordallo-Cardona et al. [18]. For this purpose, SAB agar plates were prepared with six different caspofungin concentrations (0.125, 0.25, 0.5, 1, 2, and 4 mg/L). For the exposure experiments, duplicate plates were set up to ensure reproducibility. An adjusted inoculum (100 µL) was streaked on plates containing caspofungin at 0.125 mg/L, and the plates were incubated at 37 °C for one week. The plates were visually inspected for growth every 48 h. If growth was observed, a loopful of the cultured isolates was resuspended in PBS, counted to a concentration of 1 × 10^8^ yeast/mL, and 100 µL of this fungal suspension was spread onto the next plate with a 2-fold greater concentration of caspofungin. These steps were repeated until reaching a concentration of 4 mg/L of caspofungin. The resulting evolved (or resistant) strain was named *Ch4′r*. All subsequent experiments were performed using both the wild-type (Ch4) and resistant (*Ch4′r*) strains to compare the various biological, biochemical, and virulence properties of these *C. haemulonii* strains.

### 2.3. Antifungal Susceptibility Assay

Antifungal susceptibility testing was performed following the standardized broth microdilution technique as described in the Clinical and Laboratory Standards Institute (CLSI) document M27 Ed4 [19]. The antifungals tested were anidulafungin (AND), caspofungin (CAS), and micafungin (MICA) (Sigma-Aldrich, St. Louis, MO, USA). The minimal inhibitory concentration (MIC) values of these antifungals against planktonic yeast cells were determined according to M27 Ed4 [19]. Since no breakpoints for the *C. haemulonii* species complex have been defined by the CLSI, we used the tentative breakpoints suggested for *C. auris* by the Centers for Disease Control and Prevention (CDC, Atlanta, GE, USA) since the *C. haemulonii* species complex is phylogenetically related to *C. auris* as follows: R ≥ 2 mg/L for CAS; R ≥ 4 mg/L for either AND or MICA [13].

### 2.4. Sequence Analysis of the FKS1 Gene

Genomic DNA was extracted with a Gentra^®^ Puregene^®^ Yeast and G+ Bacteria Kit (Qiagen^®^, Hilden, Germany). Hot spot (HS) regions, HS1 and HS2, were amplified with the following forward and reverse primers (1 μM concentration of each): HS1-F, 5′-TGTTCCTTTCGCTTACGCCT-3′; HS2-F, 5′-GGGTGAGCAGATGTTGTCCA-3′; HS1-R, 5′-ACGACCGATGGAGAACACAC-3′; HS2-R, 5′-CCCATGTAGATGGCGGAGTC-3′. BigDye^®^ Terminator v3.1 Cycle Sequencing Kit (Applied Biosystems, Waltham, MA, USA) was used for the sequencing reaction, precipitated with ethanol/EDTA/sodium acetate according to the protocol suggested by the manufacturer and sequenced on an ABI3730xl DNA Analyzer platform (Applied Biosystems, Waltham, MA, USA). DNA sequences and the corresponding amino acid sequences were analyzed with the SeqMan II and Bioedit 7.2 software packages (Lasergene—DNAStar, Madison, MI, USA). The sequence of the *FKS1* (orf 19.2929) gene from the *Candida* Genome Database (CGD; http://www.candidagenome.org; accessed on 10 January 2023) and from *C. haemulonii* B11899 were used as a reference [20,21].

### 2.5. Growth Curves

In this experiment, suspensions of each fungal strain were prepared at a concentration of 10^5^ yeasts/mL in SAB medium. These suspensions were then inoculated into a 96-well plate (200 μL/well) at 37 °C. The growth of the fungal strains was monitored by measuring the optical density at 540 nm using a spectrophotometer (Spectramax M2e Molecular Devices, San Jose, CA, USA) at various time points up to 96 h, which allowed for the construction of growth curve kinetics for the strains.

### 2.6. Biofilm Formation

To assess biofilm formation, fungal suspensions containing 10^6^ fungi in SAB (200 μL) were transferred to sterile 96-well polystyrene flat-bottom microplates (Costar 3599; Corning Inc., Corning, NY, USA) and then incubated without agitation at 37 °C. After 48 h, the supernatant fluids were carefully removed, and the wells were washed with saline to remove non-adherent fungal cells. Biofilm biomass quantification was performed by staining the biofilm with crystal violet after fixing it with methanol and reading at 590 nm. The metabolic activity of the biofilm was determined using a colorimetric assay, which measures the metabolic reduction of 2,3-bis(2-methoxy-4-nitro-5-sulfophenyl)-5-[(phenylamino) carbonyl]-2H-tetrazolium hydroxide (XTT; Sigma-Aldrich, St. Louis, MO, USA) to a water-soluble brown formazan product, which was measured in non-fixed biofilms at 492 nm. The quantification of the extracellular matrix in the biofilm was performed at 530 nm after safranin impregnation, using non-fixed biofilms [22]. All measurements were carried out using a microplate reader in a spectrophotometer (Spectramax M2e Molecular Devices, San Jose, CA, USA).

### 2.7. Chitin Content

To evaluate the chitin content, fungal cells (10^6^) were fixed in 4% paraformaldehyde and then stained with Calcofluor white (Sigma-Aldrich, St. Louis, MO, USA) at 5 µg/mL at room temperature for 30 min [23]. Subsequently, the yeasts were washed three times in PBS and analyzed in a flow cytometer (FacsAria Cell sorter, BD Biosciences, San Jose, CA, USA) equipped with a 15-mW argon laser emitting at 460 nm. The mapped population (10,000 events) was analyzed for fluorescence log using a single-parameter histogram. The results were expressed as the mean of fluorescence intensity (MFI).

### 2.8. Ultrastructural Architecture

For ultrastructural analysis using transmission electron microscopy (TEM), fungal cells (10^8^) were chemically fixed in a solution containing 0.1 M Na_2_HPO_4_ (pH 7.4), 2% glutaraldehyde, and 4% paraformaldehyde for 2 h at 4 °C. Following fixation, the cells were washed three times with Na_2_HPO_4_ buffer. Post-fixation was performed using 1% potassium permanganate for 1 h at 4 °C, followed by 0.15% tannic acid for 1 min at room temperature and 2% uranyl acetate for 1 h at room temperature. Dehydration of the fungal cells was carried out in a series of increasing concentrations of acetone (50, 75, 90, 95, and 100%) for 10 min at 4 °C. Infiltration was carried out at room temperature using increasing concentrations of epoxy resin (25, 50, 75, and 100%), and polymerization was performed at 60 °C for 48 h. Ultrathin sections (50 to 70 nm) were obtained using an ultramicrotome, and the sections were stained with saturated uranyl acetate and 2% lead citrate following standard procedures. Images were recorded using a 1k Gatan charge-coupled device (CCD) camera with a Tecnai 12 FEI microscope operated at 120 kV. To determine the cell wall thickness, the width of the cell wall was measured in at least 20 fungal cells from each strain using Adobe Photoshop 2021 software [24].

### 2.9. Cell Wall Integrity and Stress Response

Tests were conducted by spotting serial dilutions of fungal cells (10^4^, 10^3^, 10^2^, and 10^1^ yeasts) onto solid medium containing various well-known chemical stressors [25]. The following cell wall stressors were used: CAS (16 µg/mL), Congo red (100 µg/mL), Calcofluor white (50 µg/mL), and hygromycin B (100 µg/mL) (Sigma-Aldrich, St. Louis, MO, USA). Additionally, other stress-inducing agents were evaluated: dithiothreitol (20 mM) and tunicamycin (1 µg/mL) (Sigma-Aldrich, St. Louis, MO, USA). The plates were incubated for 2 to 4 days at 37 °C. Finally, the results of the tests were recorded by photographing the plates.

### 2.10. Macrophage Interaction

Macrophages from the RAW 264.7 cell lineage (ATCC code number TIB-71) were cultured and maintained in 25 cm^2^ culture flasks containing Roswell Park Memorial Institute 1640 (RPMI) medium (Sigma-Aldrich, St. Louis, MO, USA) supplemented with 10% fetal bovine serum (FBS; Cultilab, São Paulo, Brazil). The macrophages were grown to confluence at 37 °C in a 5% CO_2_ atmosphere. The pH of the medium was maintained at 7.2 by adding 3 g/L HEPES and 0.2 g/L NaHCO_3_ as previously described [26]. For subsequent assays, the macrophages were seeded into 24-well plates at a density of 10^5^ cells per well and incubated at 37 °C with 5% CO_2_ for 24 h in 500 µL of RPMI medium supplemented with 10% FBS. After incubation, the wells were washed with RPMI to remove non-adherent macrophages, and 500 µL of fungal suspensions prepared in RPMI without FBS (5 yeasts per macrophage) was added to each well. The initial engulfment of yeasts by the macrophages took place at 37 °C with 5% CO_2_ after 3 h. The survival/multiplication assay was evaluated after 24 h of interaction. To determine the number of intracellular yeasts, the monolayers were washed three times with PBS and then lysed with 1% Triton X-100 in PBS. The colony-forming units (CFUs) were counted to quantify the number of intracellular yeasts. Engulfment was expressed as the mean percentage of recovered yeasts relative to the number of initial yeasts in the inoculum, which was defined as 100%. Yeast survival/multiplication was expressed as the mean percentage of yeast recovered at 24 h post infection relative to the number of engulfed yeasts, also defined as 100%.

### 2.11. In Vivo Assays Using the Galleria mellonella Model

#### 2.11.1. Larval Survival Assay

The *G. mellonella* larvae were raised under controlled conditions in our laboratory, following established protocols for their entire life cycle. The procedures employed were in accordance with previously described methods until the larvae attained a weight range of 220–280 mg. Ten microliters of the standardized fungal inoculum (6 × 10^6^ yeasts) were systemically injected into *G. mellonella* larvae through the last right proleg by using a 10 μL Hamilton syringe as previously published [27]. Subsequently, the larvae were incubated at 37 °C in sterile Petri plates (10 larvae per plate). To assess survival after infection at 37 °C, the larval groups, consisting of 10 animals per group, were monitored daily for a duration of 5 days. The larvae were considered dead if they displayed a black coloration (melanization process) and showed no movement in response to touch. This visual observation was used as an indicator of larval mortality during the course of the experiment.

#### 2.11.2. Hemocyte Density

The total number of hemocytes was measured at 2 and 8 h after infection using a standardized inoculum of 6 × 10^6^ yeasts per larva. Hemolymph samples were collected by extracting 20 μL and mixing it with an equal volume of insect physiological saline buffer (IPS; 150 mM sodium chloride, 5 mM potassium chloride, 10 mM Tris-HCl, pH 6.9, 10 mM EDTA, and 30 mM sodium citrate) [28]. The collected hemolymph samples were then further diluted 1:10 in IPS. Hemocyte counts were performed using a Neubauer chamber [29], and the results were expressed as hemocytes/mL.

#### 2.11.3. Larvae Treatment with Antifungals

Thirty minutes after infecting the larvae with 6 × 10^6^ yeasts, the larvae were treated with 10 µL of echinocandins (CAS or MICA) injected into the last left proleg. The larvae were then placed in sterile Petri plates and incubated at 37 °C. The following groups were included in the evaluation: untreated control (larvae that did not receive any injection), DMSO (larvae inoculated with a vehicle solution containing 5% DMSO in PBS), toxicity drug control (non-infected larvae inoculated with each antifungal), negative control (larvae inoculated with yeasts and treated with PBS), and positive controls (larvae inoculated with yeasts and treated with each antifungal drug). The concentrations of the administered antifungals corresponded to the recommended dose of CAS for treating invasive candidiasis in humans [30]. The figures were abbreviated, and both the PBS control group and the toxicity drug control group results are not presented as they did not have any significant impact on larval survivability.

#### 2.11.4. Fungal Burden

To evaluate the impact of echinocandins on yeast load in the hemolymph, groups of five larvae were infected with 6 × 10^6^ yeasts per larva. Subsequently, the larvae received a single dose of CAS or MICA at 30 min post infection. At time 0 h (immediately after the antifungal injection) and at 48 h post treatment, 20 µL of hemolymph was collected from each larva, diluted 1:10 in IPS buffer, and then subjected to serial dilutions in SAB. The diluted samples were plated onto SAB agar plates supplemented with 40 mg/L of streptomycin (Sigma-Aldrich, St. Louis, MO, USA). The agar plates were incubated at 37 °C for 48 h, and CFUs were enumerated to quantify the yeast load [27].

### 2.12. Statistical Analysis

All assays were performed in triplicate in at least three independent experiments. Statistical analyses were performed by one-way analysis of variance (ANOVA). All analyses were carried out using GraphPrism, version 7. Differences were considered significant at *p* < 0.01. Differences in the survival of *G. mellonella* larvae were determined using the Kaplan–Meier method, and the log-rank test was used to compare the survival curves.

## 3. Results

### 3.1. Generation and Altered Susceptibility in the Evolved C. haemulonii Strain

Prior to the directed evolution experiment, the susceptibility of the *C. haemulonii* strain Ch4 to three clinically available echinocandins (AND, CAS, and MICA) was determined. The MIC values for CAS, MICA, and AND were found to be 0.5 mg/L, 0.25 mg/L, and 0.125 mg/L, respectively (Table 1). In the in vitro experiment, exposing *C. haemulonii* cells to a direct lethal dose of CAS did not lead to the emergence of resistant cells. However, in the progressive evolution experiment, it was observed that some fungal cells were able to survive and form colonies on plates containing sub-inhibitory concentrations of CAS. This observation suggests that increasing the CAS concentrations in subsequent experiments was indeed effective in selecting and enriching resistant populations of *C. haemulonii*. The fungal colonies that showed enhanced growth in different concentration plates were further assessed for responsiveness in the presence of AND, CAS, and MICA. The parental isolate (Ch4), when grown on plates containing CAS at concentrations ranging from 0.25 mg/L to 4 mg/L, developed pan-echinocandin resistance (Table 1). One noteworthy observation is that isolates cultivated in the presence of lower CAS concentrations (0.25 mg/L and 0.5 mg/L) were considered merely tolerant to echinocandins since their MIC values remained equal to those of the parental strain. The emergence of a resistance profile was only evident after repeated exposure to CAS at a concentration of ≥1 mg/L (Table 1). The *C. haemulonii* strain resistant to echinocandins was designated as *Ch4′r*, which was obtained through selection in the presence of CAS at 4 mg/L. Both the parental (Ch4) and resistant (*Ch4′r*) strains were utilized in subsequent experiments to evaluate their phenotypic and genotypic properties.

### 3.2. FKS1 Gene Analysis

In both *C. albicans* and non-*albicans Candida* species, mutations associated with echinocandin resistance have been identified within two specific regions of the *FKS1* gene, commonly referred to as “hot-spots” 1 and 2 (HS1 and HS2, respectively) [4]. In the case of *C. auris*, these “hot-spots” are located between amino acids 641–649 and 1345–1365 in the Fks1 protein [31]. Our study’s findings revealed that the *C. haemulonii* strain *Ch4′r* displayed a single nucleotide substitution in the *FKS1* gene at position 4061 within the HS2 region. This substitution led to a purine modification, changing G to A. Further sequencing of the *FKS1* gene confirmed that the *Ch4′r* strain exhibited an arginine–histidine amino acid substitution (R1354H) at the corresponding position to the characterized R1354 in the HS2 region of the *FKS1* gene in *C. auris* (Table 2). This observation substantiates its echinocandin-resistant profile.

### 3.3. Cell Wall Integrity and Stress Response

**Figure 1:**
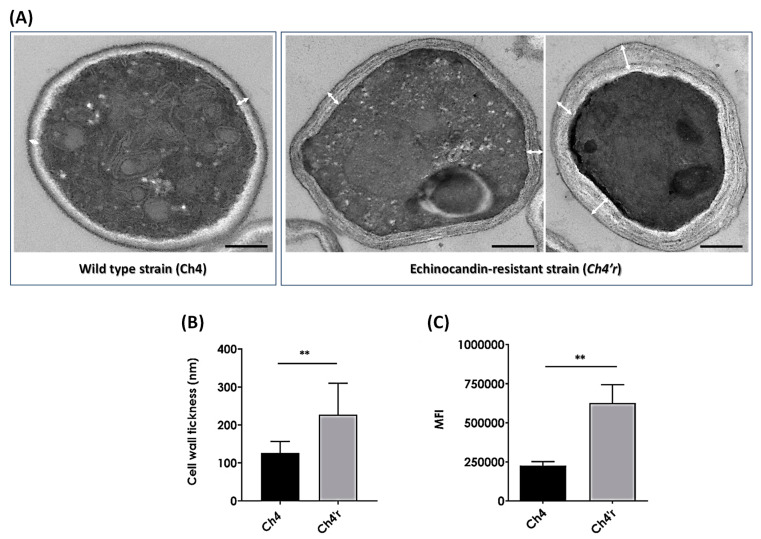
(**A**) Transmission electron microscopy of ultrathin sections. Representative images of a yeast cell from *C. haemulonii* strains Ch4 (parental or wild-type strain) and *Ch4′r* (mutant or resistant strain). The scale bars correspond to 0.5 µm. The white arrows show the cell wall thickness. (**B**) Cell wall thickness from parental and mutant strains of *C. haemulonii* by analyzing the TEM images. (**C**) Level of chitin from both strains by using Calcofluor white staining and flow cytometry analysis. The results are expressed as the mean of fluorescence intensity (MFI). The results are expressed as the mean ± standard deviation. The symbols (**) indicate *p*-values of <0.01, as determined using Student’s *t* test.

In this series of experiments, our objective was to examine whether the development of resistance through CAS exposure would have an impact on cell wall rearrangement. Firstly, we conducted TEM analysis in order to observe the ultrastructure of the cell wall. The results demonstrated that the cell wall remained intact in both parental and mutant strains of *C. haemulonii*. As shown in Figure 1A, the cell wall of the parental strain was composed of two main layers that had different electron densities. As described in previous studies, the inner layer comprises a relatively conserved structural skeleton rich in chitin and glucan, while the outer layer consists of heavily mannosylated glycoproteins with modified *N*- and *O*-linked oligosaccharides, which are fibrils associated with adhesion [32]. In contrast, the *Ch4′r* mutant strain exhibited a significant proportion of cells with a thickened cell wall (Figure 1B). Moreover, in comparison with the parental strain, the cell wall in the mutant strain appeared more homogeneous, and the cytoplasmic content exhibited a higher electron density, indicative of cellular integrity (Figure 1A). To further investigate the alterations in cell wall components, we quantified the levels of chitin in both the parental and mutant strains. The results revealed that the resistant strain *Ch4′r* displayed elevated chitin levels, 2.5 times higher than those in the parental strain (Figure 1C).

**Figure 2:**
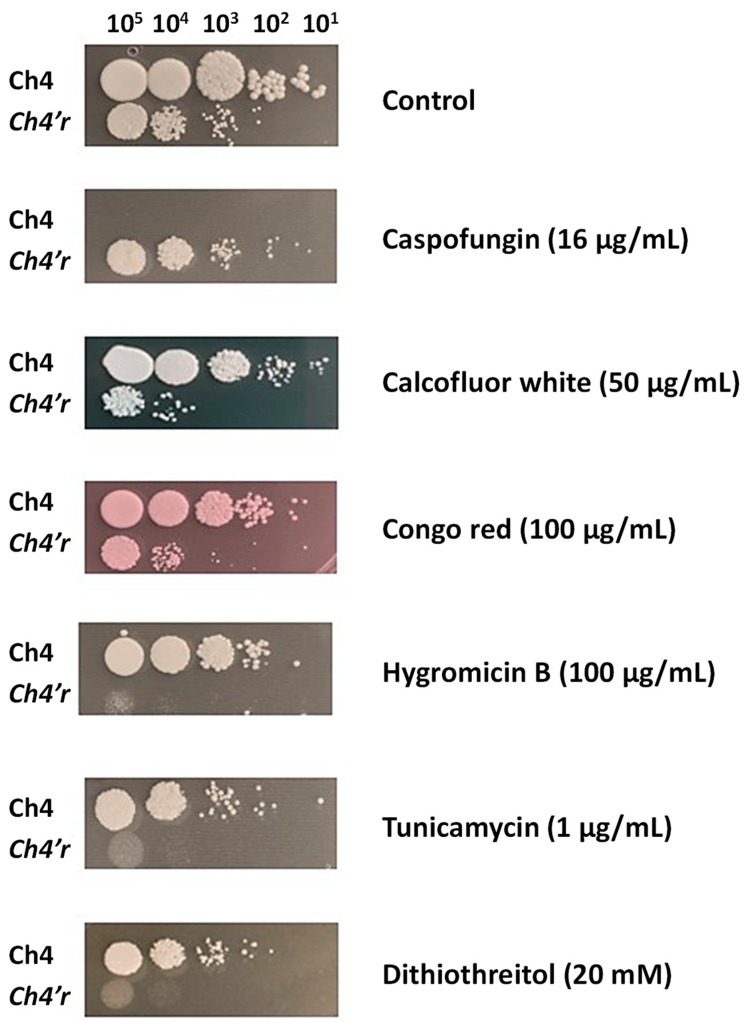
Representative images of the growth capabilities of parental (Ch4) and resistant (*Ch4′r*) strains of *C. haemulonii* on SAB agar with different cell stressors added. From left to right, 10^5^, 10^4^, 10^3^, 10^2^, and 10^1^ yeasts were spotted on each plate containing different stressors and incubated at 37 °C for 2 to 4 days. Control plates containing just SAB were incubated for 2 days.

Since the cell wall components were rearranged differently, we further evaluated the fungal integrity by conducting a spot plate assay using SAB agar supplemented with various stress-inducing agents. As expected, the parental strain of *C. haemulonii* was unable to grow in medium supplemented with CAS, unlike the resistant strain (Figure 2). Conversely, the resistant strain showed greater sensitivity to the presence of cell wall stressors such as Calcofluor white, Congo red, and hygromycin B. Additionally, the resistant strain exhibited hypersensitivity to growth in the presence of tunicamycin and dithiothreitol, which are endoplasmic reticulum stressors that lead to the accumulation of unfolded proteins (Figure 2). These results collectively emphasize that the increase in echinocandin tolerance/resistance is accompanied by cell wall restructuring.

### 3.4. Acquisition of Resistance to CAS Is Associated with a Fitness Cost

**Figure 3:**
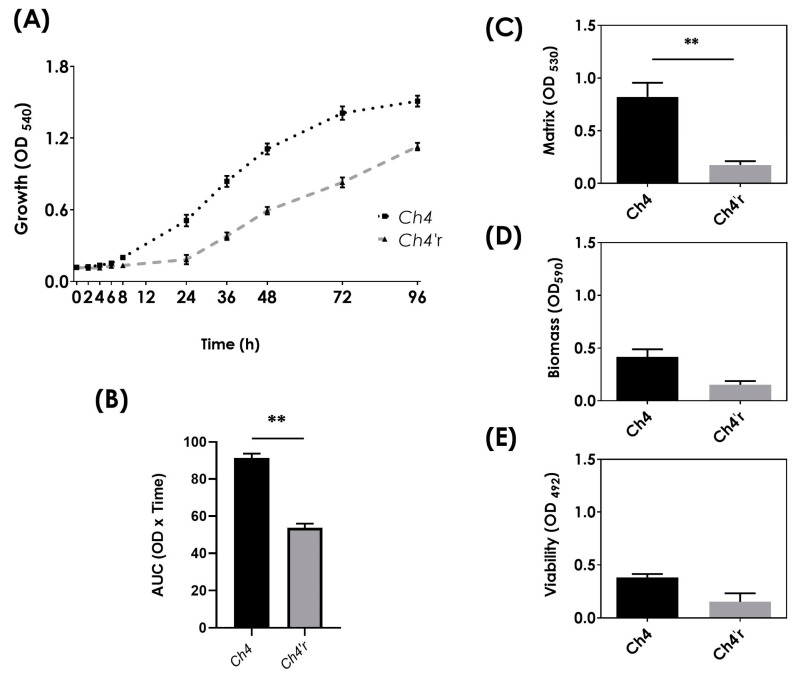
Growth proliferation and biofilm formation in parental (Ch4) and CAS-resistant (*Ch4′r*) strains of *C. haemulonii*. (**A**) The graphic shows the yeast growth curves in SAB at 37 °C for a period of 96 h. (**B**) Area under the curve (AUC) values corresponding to the plotted growth curves. (**C**–**E**) Biofilm formation on the polystyrene surface: fungal biomass, evidenced by the incorporation of crystal violet at 590 nm (**C**), extracellular matrix, evidenced by safranin at 530 nm (**D**), and metabolic activity, evidenced by XTT at 492 nm (**E**). The results are expressed as the mean ± standard deviation. Statistical differences were highlighted between parental and resistant strains by student’s *t* test (**, *p* < 0.01).

Considering that the CAS-resistant strain of *C. haemulonii* exhibited a distinct cell wall ultrastructure (Figure 1) and a different ability of growth in solid medium containing various cell stressors (Figure 2), we conducted measurements of growth kinetics over time and assessed the strain’s ability to form a biofilm structure on a polystyrene surface in order to determine whether the evolution of CAS resistance was associated with a fitness cost. The growth curves showed that the parental strain Ch4 had a faster growth rate compared to the CAS-resistant strain *Ch4′r* (Figure 3). Similarly, the biofilm formed by the resistant strain was significantly reduced when compared to the parental strain, as indicated by the evaluation of three biofilm parameters: biomass, viability, and extracellular matrix (Figure 3C–E).

### 3.5. Fungi–Macrophage Interplay

**Figure 4:**
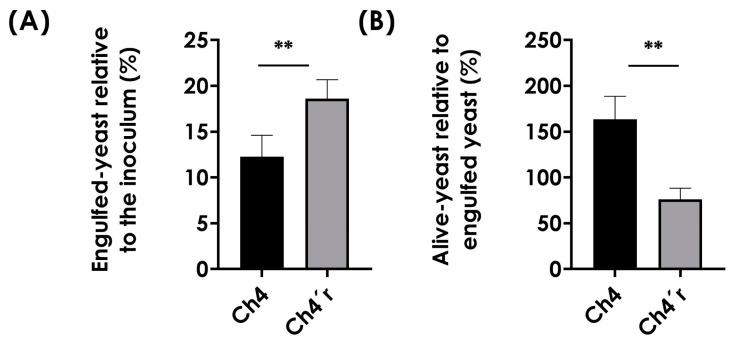
Interaction of RAW 264.7 macrophages with *C. haemulonii* parental type strain Ch4 and resistant strain *Ch4′r*. (**A**) Phagocytosis was expressed as the number of associated yeasts 3 h after infection in relation to the number of initial inoculum. (**B**) Yeast survival/multiplication was expressed as the number of associated yeasts 24 h after infection versus the number obtained after counting the 3 h period. The values represent the mean ± standard deviation (*n* = 3). Statistical differences are highlighted the between strains by Student’s *t* test (**, *p* < 0.01).

Since the organization and composition of the fungal cell wall are crucial for the interaction and recognition of yeasts by the host immune system, we decided to study this interface. The assessment of the interaction with macrophages revealed that the engulfment of yeasts from the CAS-resistant strain of *C. haemulonii* by murine RAW 264.7 macrophages was greater compared to the parental cells (Figure 4A). In contrast, the parental strain exhibited higher resistance to macrophage killing compared to the resistant strain, as indicated by the number of viable fungal cells after 24 h of interaction (Figure 4B).

### 3.6. Acquisition of CAS Resistance Resulted in In Vivo Attenuated Virulence and Antifungal Resistance

**Figure 5:**
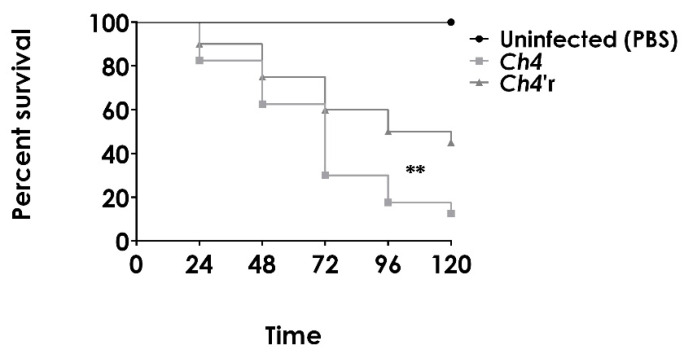
In vivo virulence of both *C. haemulonii* parental Ch4 and resistant *Ch4′r* strains in the *G. mellonella* larvae model. The results are expressed as the percentage of survival compared to uninfected (PBS-treated) larvae. Survival analyses were determined using the log-rank test and Kaplan–Meier survival curves. Statistical differences between the strains are highlighted (**, *p* < 0.01).

To confirm the attenuated virulence of the CAS-resistant strain, we conducted a test using *G. mellonella* larvae as a model for disseminated candidiasis. In this sense, the susceptibility of *G. mellonella* was assessed by monitoring the survival rate of larvae over a period of 5 days at 37 °C after infecting them with both the *C. haemulonii* parental strain Ch4 and the CAS-resistant strain *Ch4′r*. The results clearly demonstrated that the survival rate of larvae infected with the resistant strain *Ch4′r* was significantly higher compared to those inoculated with the parental strain (Figure 5). It is important to note that no deaths were observed in larvae that were not infected or injected with PBS throughout the entire duration of the experiment.

**Figure 6:**
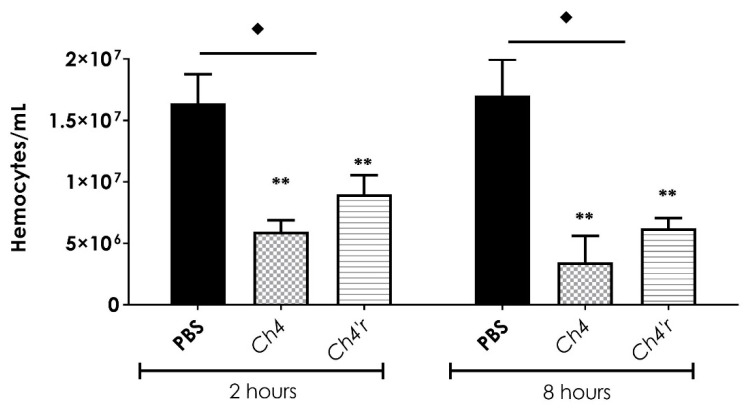
*Galleria mellonella* larvae’s response to infection by the parental Ch4 and resistant *Ch4′r* strains of *C. haemulonii* was examined to assess the modulation of the immune response. To complete this, the overall hemolymph hemocyte density was measured at 2 and 8 h after infection with a fungal load of 6 × 10^6^ fungi per larva. Statistical differences between the control and strains were highlighted and analyzed by two-way ANOVA with the Bonferroni post-test (♦, *p* < 0.01 between the control and strains and ** *p* < 0.01 between both fungal strains).

Subsequently, we investigated whether the different strains of *C. haemulonii* had any impact on modulating the immune response by altering the density of *G. mellonella* hemocytes. As depicted in Figure 6, both the parental and the resistant *C. haemulonii* strains resulted in a decrease in hemocyte density when compared to the non-infected group (larvae inoculated with only PBS, control group). However, the parental strain exhibited a significantly greater reduction in the number of hemocytes, as observed at 2 and 8 h after infection, compared to the resistant strain (Figure 6).

**Figure 7:**
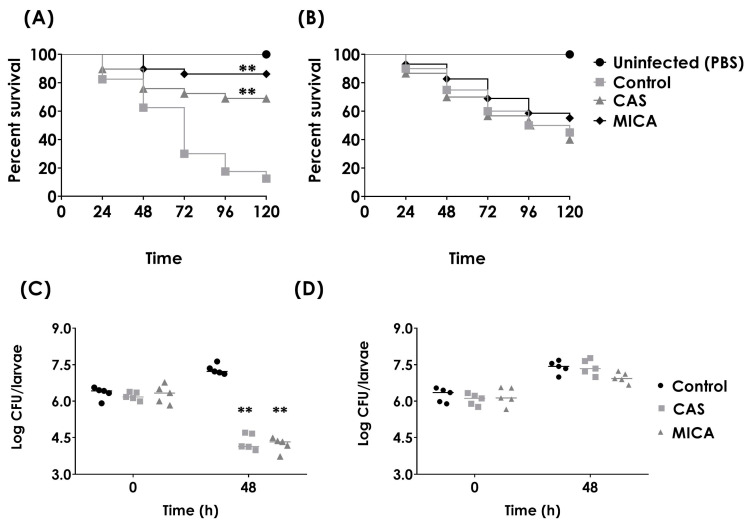
In vivo antifungal susceptibility profiles of parental and resistant strains of *C. haemulonii* in the *G. mellonela* model. (**A**,**B**) Efficacy of CAS and MICA (both at 1 mg/kg) during parental Ch4 (**A**) and resistant *Ch4′r* (**B**) infections at a final concentration of 6 × 10^6^ yeasts/larva. The larvae were incubated at 37 °C for up to 5 days. Statistical differences between the treatment and control are highlighted (**, *p* < 0.01). (**C**,**D**) Effect of antifungal treatment of CAS and MICA on fungal burden during parental Ch4 (**C**) and resistant *Ch4′r* (**D**) infections in treated or untreated *G. mellonella* (control) at a final concentration of 6 × 10^6^ yeasts/larva. The results are expressed as the Log CFU/larva, and the horizontal bars represent the median value of larval load per group (5 larvae). Statistical differences were highlighted (**, *p* < 0.01) for the group of larvae that were infected and received only PBS at the same time.

To assess whether the in vitro antifungal susceptibility profile correlated with the in vivo profile, infected larvae (6 × 10^6^ yeasts/larva) were treated with CAS (1 mg/kg) or MICA (1 mg/kg) (Figure 7). The results showed that larvae infected with the parental strain and subsequently treated with CAS or MICA exhibited protection from the infection and a reduced fungal burden compared to untreated larvae (Figure 7A,C). Conversely, the resistant strain *Ch4′r* maintained its resistance profile, as treatment with CAS or MICA did not enhance larval survival or reduce the fungal load (Figure 7B,D).

## 4. Discussion

Infections caused by species of the *C. haemulonii* complex have shown limited response to azoles and AMB, as mentioned earlier. Therefore, treatment with echinocandins is often recommended as a first-line approach. However, it is important to note that resistance to echinocandins can develop during therapy, primarily due to repeated or prolonged drug exposure, although resistance can also emerge after brief exposure [1]. Nevertheless, some reports have indicated specific clinical isolates with disproportionately high MIC values to echinocandins compared to the current literature [33,34,35]. Up to this moment, no established breakpoints for echinocandins (or any other antifungal class) have been proposed for the *C. haemulonii* species complex. On the other hand, proposed echinocandin breakpoints for *C. auris* were recently published on the Centers for Disease Control and Prevention (CDC, Atlanta, GE, USA) website (https://www.cdc.gov/fungal/candida-auris/c-auris-antifungal.html; accessed on 18 January 2023), as follows: the resistant breakpoint for CAS is ≥2 mg/L, and for both MICA and AND, it is ≥4 mg/L. Based on these statements, in the present study, we adopted the breakpoints proposed for *C. auris* as a standard to compare with the *C. haemulonii* complex since the latter is composed of species phylogenetically related to *C. auris*. The ability of *C. haemulonii* to rapidly acquire resistance to echinocandins is highly concerning, particularly considering that echinocandins are often used as the primary treatment option for MDR *Candida* species. This development poses a significant challenge as it potentially eliminates an effective therapeutic option for treating infections caused by these species.

Previous studies have demonstrated that acquired resistance to echinocandins in clinical isolates of *Candida* can result in fitness costs or conditioning effects that lead to decreased virulence [36,37,38,39]. Similarly, in our study, we observed that the CAS-evolved resistant strain exhibited reduced virulence in vivo, as evidenced by higher survival rates of *G. mellonella* larvae compared to the parental strain. In the context of in vitro interactions, the survival of *C. haemulonii* yeasts challenged by macrophages may depend not only on their intracellular survival but also on the efficiency of phagocytosis. It is known that fungal β-glucan masking by mannan can inhibit fungal recognition and killing by macrophages [40,41]. In our resistant strain, we observed more efficient phagocytosis by macrophages, suggesting a rearrangement of fungal cell wall components responsible for the recognition and initial interaction between these two cell types. We hypothesize that CAS treatment may enhance the immunogenicity of the resistant strain by unmasking the β-glucan layer. The exposure of the glucan layer plays a crucial role in the detection of *C. albicans* [42] and also in the related strain *C. auris* [43].

The cell wall remodeling of the *C. haemulonii* resistant strain *Ch4′r* was evident from its inability to form dense biofilms and grow in the presence of either cell wall-damaging or stress-inducing agents. Biofilm formation is an important virulence trait in pathogenic yeasts belonging to the *Candida* species [44]. Ibe and Munro [45] recently reviewed the connections between cell wall integrity and stress-activated pathways that regulate cell wall remodeling and the expression of certain mannoproteins that may be involved in *Candida* biofilm formation. Although further studies are required to understand CAS-induced cell wall stress responses in other *Candida* species, available data suggest similarities in signaling pathways, the response strategies deployed, and the surface-located proteins involved in maintaining cell wall integrity.

The understanding of how the inhibition of cell wall synthesis is coordinated with other cellular responses remains limited. Echinocandins induce a set of genes from the cell integrity signaling pathway mediated by protein kinase C (PKC), along with other genes involved in cell wall architecture and maintenance [46,47]. In *Candida* species, when exposed to echinocandins, there is a compensatory increase in chitin synthesis that helps maintain the structural integrity of the cell wall. This increase serves as a short-term adaptive strategy for fungal cells before the development of Fks1/Fks2-mediated resistance mechanisms [48,49,50]. Conversely, inhibiting chitin biosynthesis using nikkomycin Z, a chitin synthase inhibitor, makes *Candida* cells more susceptible to CAS treatment [51]. CAS treatment can either increase the exposure of β-(1,3)-glucan on the cell surface or cause its depletion. Studies have shown that the increased exposure of β-(1,3)-glucan, induced by CAS treatment or as a result of resistance, enhances the elimination of fungal cells by phagocytes through receptors like Dectin-1, both in vitro and in vivo models of infection [39,42,52,53]. However, other studies have demonstrated that the absence of β-(1,3)-glucan can also trigger an effective antifungal immune response by activating dendritic cells and complement [54]. Notably, in the CAS-resistant *C. haemulonii* strain *Ch4′r*, significant changes in chitin levels were observed in the cell wall compared to the parental strains. Recent findings by Zamith-Miranda et al. [55] further support compensatory mechanisms employed by *C. auris* to overcome the absence of glucans. These mechanisms involve altered transcript levels and the modified detection of mannans and chitin in the cell wall when exposed to high concentrations of CAS. Further investigations are warranted to delve into the intricate interplay of cell wall components and stress mechanisms. Our current report is constrained by its focus on a single evolved strain, highlighting the need for additional studies to provide a more comprehensive understanding.

The positions and types of mutations in the *FKS* gene vary among different *Candida* species and have a selective impact on the susceptibility to echinocandin treatment, both in vitro and in vivo [3,31]. Amino acid substitutions in the Fks subunits can lead to significantly higher MIC values (10- to 100-fold) and reduce the sensitivity of glucan synthase to the drugs by up to 3000-fold [27,56,57]. In *C. auris*, these “hot-spots” are situated between amino acids F635 to P643 and D1350 to L1357 within the Fks1 protein [58]. Notably, significant mutations in the *FKS* genes have been linked to inadequate pharmacodynamic responses and diminished clinical treatment outcomes [59,60]. It is important to emphasize that *C. haemulonii*, similar to other MDR *Candida* species like *C. auris* and *C. glabrata*, is a haploid organism. In our study, we identified an in vitro evolved resistant strain harboring an R1354H mutation within hot spot region 2 of the Fks1 protein. The initial documentation of this mutation was reported by Asadzadeh et al. [61] in isolates of *C. auris* derived from a candidemia patient. Recently, Kiyohara et al. [62] also conducted sequencing on a CAS-resistant clinical isolate (clade I) and identified the identical resistance mutation (G4061A leading to R1354H). These researchers further validated through the (CRISPR)-Cas9 editing system that the introduction of this mutation into a wild-type strain induced CAS resistance. Furthermore, they demonstrated that *C. auris* R1354H mutants displayed CAS resistance in an in vivo model. Our data substantiate the fact that the sole presence of the R1354H mutation in *C. haemulonii* is indeed capable of conferring in vivo resistance against both CAS and MICA in the *G. mellonella* model.

## 5. Conclusions

Taken together, these data reveal a direct connection between acquired resistance to echinocandins and attenuated virulence in *C. haemulonii*, highlighting the importance of further understanding the biology of clinically relevant MDR fungi. The accurate diagnosis of emerging, rare, and resistant species, along with the surveillance of fungal diseases, is crucial to improving our understanding of their epidemiology and contributing to precise clinical treatment options and successful outcomes.

## Figures and Tables

**Table 1:** MIC values determined for the parental (Ch4) and resistant (*Ch4′r*) strains of *C. haemulonii*.

Antifungals		MIC Values (mg/L)				
	Parental Strain	Evolved Strains Obtained under Different CAS Concentrations ^a^				
	Ch4	*Ch4* 0.25 mg/L	*Ch4* 0.5 mg/L	*Ch4′r* 1.0 mg/L	*Ch4′r* 2.0 mg/L	*Ch4′r* 4.0 mg/L
**CAS**	0.5	0.5	0.5	>16	>16	>16
**MICA**	0.25	0.25	0.5	>16	>16	>16
**AND**	0.125	0.125	0.25	>16	>16	>16

^a^ Colonies recovered from each CAS concentration used on plates to select resistance.

**Table 2:** Sequence of the *FKS1* gene and the Fks1 protein in parental (Ch4) and resistant (*Ch4′r*) *C. haemulonii* strains.

Genes	Nucleotide Sequence (Position/Mutation)	Amino Acid Sequence (Position/Mutation)	Fungal Strains
***FKS1* HS1**			
	1924—TTCTTGACTTTGTCCTTGAGAGATCCT	635—FLTLSLRD	B11899 ^a^
	1924—TTCTTGACTTTGTCCTTGAGAGATCCT	635—FLTLSLRD	Ch4
	1924—TTCTTGACTTTGTCCTTGAGAGATCCT	635—FLTLSLRD	*Ch4′r*
***FKS1* HS2**			
	4048—GACTGGATTAGACGTTATACCTTG	1350—DWIRRYT	B11899 ^a^
	4048—GACTGGATTAGACGTTATACCTTG	1350—DWIRRYT	Ch4
	4048—GACTGGATTAGACATTATACCTTG	1350—DWIRHYT	*Ch4′r*

^a^ Sequence of *C. haemulonii* strain B11899 [21]. Red letters represent the altered nucleotide and amino acids detected in the resistant strain.

## Data Availability

Not applicable.
